# Effects of whole maize high-grain diet feeding on colonic fermentation and bacterial community in weaned lambs

**DOI:** 10.3389/fmicb.2022.1018284

**Published:** 2022-12-08

**Authors:** Chao Cheng, Yuyang Yin, Gaorui Bian

**Affiliations:** ^1^College of Animal Science and Food Engineering, Jinling Institute of Technology, Nanjing, China; ^2^College of Animal Science and Technology, Nanjing Agricultural University, Nanjing, China; ^3^Huzhou Academy of Agricultural Sciences, Huzhou, China

**Keywords:** lambs, maize, colonic content, colonic mucosa, bacterial community

## Abstract

High-grain diet is commonly used in intensive production to boost yield in short term, which may cause adverse effects such as rumen and colonic acidosis in ruminants. Maize is one of the key components of high-grain diet, and different processing methods of maize affect the digestive absorption and gastrointestinal development of ruminants. To investigate the effects of maize form in high-grain diets on colonic fermentation and bacterial community of weaned lambs, twenty-two 2.5-month-old healthy Hu lambs were fed separately a maize meal low-grain diet (19.2% grain; CON), a maize meal high-grain diet (50.4% grain; CM), and a whole maize high-grain diet (50.4% grain; CG). After 7 weeks of feeding, the total volatile fatty acid concentration (*P* = 0.035) were significantly higher in lambs from CM than that from CON. The sequencing results of colonic content microbial composition revealed that the relative abundance of genera *Parasutterella* (*P* = 0.028), *Comamonas* (*P* = 0.031), *Butyricicoccus* (*P* = 0.049), and *Olsenella* (*P* = 0.010) were higher in CM than those in CON; compared with CM, the CG diet had the higher relative abundance of genera *Bacteroides* (*P* = 0.024) and *Angelakisella* (*P* = 0.020), while the lower relative abundance of genera *Olsenella* (*P* = 0.031) and *Paraprevotella* (*P* = 0.006). For colonic mucosal microbiota, the relative abundance of genera *Duncaniella* (*P* = 0.024), *Succiniclasticum* (*P* = 0.044), and *Comamonas* (*P* = 0.012) were significantly higher in CM than those in CON. In comparison, the relative abundance of genera *Alistipes* (*P* = 0.020) and *Campylobacter* (*P* = 0.017) were significantly lower. And the relative abundance of genera *Colidextribacter* (*P* = 0.005), *Duncaniella* (*P* = 0.032), *Christensenella* (*P* = 0.042), and *Lawsonibacter* (*P* = 0.018) were increased in the CG than those in the CM. Furthermore, the CG downregulated the relative abundance of genes encoding infectious-disease-parasitic (*P* = 0.049), cancer-specific-types (*P* = 0.049), and neurodegenerative-disease (*P* = 0.037) in colonic microbiota than those in the CM. Overall, these results indicated that maize with different grain sizes might influence the colonic health of weaned lambs by altering the composition of the colonic bacterial community.

## Introduction

In the current intensive farming system, feeding ruminants a high-grain diet is extensively used to boost yield and earn immediate financial gains (Plaizier et al., 2008). However, the high proportion of starch and low level of fiber in high-grain diets can lead to more volatile fatty acids (VFAs) and lipopolysaccharide in the colon due to increased carbohydrate fermentation in the post-rumen. Then it was followed by a decrease in colonic pH and further changes of microbiota in the colon and injuries to the colonic epithelium (Metzler-Zebeli et al., 2013; Wang et al., 2017).

The ruminant colonic epithelium exists as a physical and immunological barrier to protect animal health (Kostic et al., 2013; Steele et al., 2016). Meanwhile, the microbial communities associated with colonic content play an important role in nutrient digestion of ruminants. It was reported that the nitrogen digestion and VFAs production by microbiota in the large intestine of ruminant can account for up to 7 and 17% of the total intestine (Hoover, 1978; Vanhatalo and Ketoja, 1995). Furthermore, there are remarkable differences between the microbiota of colonic contents and mucosa in pre-weaned calves, suggesting that they may serve different functions (Malmuthuge et al., 2014), and the gastrointestinal mucosa-associated microbiota is believed to be vital for regulating barrier function and the immune system (Hooper et al., 2012). Colonic fermentation and immunity are crucial for the growth and health of ruminants, so it is essential to prevent or mitigate the adverse effects of high-grain diets on the colonic content or mucosal microbiota through nutritional strategies.

Maize, the main grain component of feed (Humer and Zebeli, 2017), can be processed by physical, chemical, and biological methods. Callison et al. (2001) demonstrated with primiparous Holstein cows that the digestibility of nonstructural carbohydrates in the rumen increased when the size of the corn crush was decreased. Chester-Jones et al. (1991) found that feeding whole corn to weaned calves tended to increase the average daily gain (ADG) and feed conversion ratio (FCR). Lopez et al. (2014) demonstrated that whole maize high-grain diets had beneficial effects on the metabolic energy of goats. These studies have shown that feeding larger particle sizes of grains facilitates the promotion of nutrient digestion and ruminant performance, which might be related to the beneficial fermentation of the microbial community under these conditions in the digestive tract. Unfortunately, limited studies have focused on the effects of maize forms on the colonic microbial community and mucosal morphology structure in fattening lambs.

Studies have shown that the entire digestive tract is not fully developed in weaned lambs (Kelly and Coutts, 2000). Early nutritional intervention can substantially impact their intestinal mucosal morphology and microbial composition (Abecia et al., 2013; Yanez-Ruiz et al., 2015), which will further influence their lifelong health and performance (Jiao et al., 2016). Therefore, we hypothesized that replacing crushed maize with whole maize could alleviate the deleterious effects of high-grain diets on developing ruminant colon in this study. To test the hypothesis, the colonic epithelial morphology and microbial community were examined in weaned lambs reared in a maize meal low-grain diet, maize meal high-grain diet, and whole maize high-grain diet.

## Materials and methods

### Experimental animals and feeding management

The animal experiment was approved by the Animal Protection and Use Committee of Nanjing Agricultural University [Authorisation SYXK(Su)2019-0074] and was performed following the Regulations for the Administration of Affairs Concerning Experimental Animals (the State Science and Technology Commission of P. R. China, 1988). The study was conducted at the experimental station located at Nanjing Agricultural University, Jiangsu Province, China, with twenty-two healthy 2.5-month-old male lambs being used and randomly allocated to three groups: one group was fed a maize meal low-grain diet (19.2% grain; CON, *n* = 7), another group was fed a maize meal high-grain diet (50.4% grain; CM, *n* = 8), and the other group was fed a whole maize high-grain diet (50.4% grain; CG, *n* = 7). The formulas of lambs were designed according to the feeding standard of NY/T 816-2004 fattening lambs (MOA, 2004), and the crude protein, starch, neutral detergent fiber, acid detergent fiber, and crude ash were measured using standard methods of AOAC (2007). Components and nutrient compositions of the diets can be found in Supplementary Table 1. The maize meal was directly crushed by hammer mill without sieve, with an average grain size of 2.57 mm; whole maize was fed directly without processing. The total experimentation period lasted 49 days after a 7-day acclimatization phase. At 8:00 and 17:00 each day for the course of the trial, all lambs were provided water and food. Food intake was recorded daily, and weight changes were monitored weekly.

### Sample collection

On day 50, all lambs were slaughtered 4 h after the morning feeding. Colon samples were separated with a blunt instrument immediately after evisceration. The pH of the representative colonic fluid was measured using a portable pH meter (HI 9024C; HANNA Instruments, Woonsocket, RI, USA). Ten g samples of the content were mixed with a double amount of distilled water and immediately centrifuged at 2,000 x *g* for 10 min and stored at −20°C for analysis of VFAs (GC-14B, Shimadzu, Japan). The colon content was collected and stored in liquid nitrogen for subsequent microbial DNA extraction. Meanwhile, a section of colonic tissue was collected and washed three times in ice-cold phosphate-buffered saline solution immediately (within 5 min) after slaughter. Then, the washed colon tissue was divided into two parts. The first part of the sample was cut into approximately 2 × 2 cm and the mucosal tissue scraped with sterile sections before being stored in liquid nitrogen until microbial DNA extraction. And samples from the second part were immediately fixed in 4% paraformaldehyde (Sigma, St. Louis, MO, USA) for histomorphometric microscopic analysis.

### Determination of fermentation parameters

The concentration of VFAs in colonic content was measured by capillary column gas chromatography (GC-14B; Shimadzu company; Japan) (Qin, 1983). One gram of sample was taken to the centrifuge tube, and 5–10 times of double-distilled water was added and mixed thoroughly. Then 0.2 ml of 25% (w/v) metaphosphoric acid was added to 1 mL of supernatant and stored overnight at −20°C. After thawing, we centrifuged the sample at 12,000 rpm for 10 min and retained the supernatant, which was then centrifuged at 12,000 rpm for another 10 min before measurement. The relative correction factors of organic acids could be calculated from the respective peak area of standard samples and internal standard crotonic acid. The concentrations andproportions of VFAs in each sample could be calculated according to the weight (or concentration) of VFAs in proportion to their peak area.

### Histological measurements

The colonic tissues were embedded with paraffin and then sliced (6 μm) and stained with hematoxylin and eosin. The microscopist was blinded to the treatment conditions during histomorphometric analysis. Three slides were prepared for each lamb, and two images were taken for each slide. The tissues were fixed with 2.5% glutaraldehyde for at least 24 h, post-fixed in 1% osmium, and embedded in Epon Araldite. Semi-thin sections (0.25–0.5 μm) and ultrathin sections (70–90 nm) were cut using a glass cutter. Then the semithin sections were stained with 1% toluidine blue and 1% sodium borate before the ultrathin sections were stained with uranyl acetate and lead citrate. We used a transmission electron microscope (H-7650; Hitachi Technologies, Tokyo, Japan) to determine the ultrastructures of the colonic epithelial cells.

### Microbial DNA extraction

The colonic content and mucosa tissue samples were processed separately, and the microbial DNA was extracted using the DNA Extraction Kit (Mobio laboratory, Carlsbad, CA, USA). The concentration and purity of the extracted DNA samples were measured by the Nanodrop 2000 spectrophotometer (Thermo Fisher Scientific, USA), ensuring that the OD260/280 value was between 1.80 and 2.00. Then the DNA samples were stored at −80°C for subsequent sequencing analysis.

### Sequencing data processing of bacterial 16S rRNA gene

Bacterial 16S rRNA gene sequences were amplified by forwarding primer (5’-CCA TCT CAT CCC TGC GTG TCT CCG ACT CAG-3’) and reverse primer (5’-CCT ATC CCC TGT GTG CCT TGG CAG TCTCAG-3’). Sample identification in the forwarding primer was a unique 12-base barcode. The PCR reaction was performed in a 50 μL C1000 thermal cycler (Bio-Rad, USA) with 10 μL 5x Fast Pfu buffer, 50 ng of DNA, 0.4 μM of each primer, 0.5U FastPfu polymerase, and 2.5 mM dNTPs. The PCR amplification process was as follows: preliminary denaturation at 95°C for 2 min; cycles (30 times for bacteria/archaea/protozoa; 35 times for fungi) of denaturation (95°C, 20 s); annealing (60°C for bacteria/58°C for archaea/54°C for protozoa/50°C for fungi, 20 s) and elongation (65°C, 1 min); and a final extension at 65°C for 7 min (Mao et al., 2012; Kittelmann et al., 2013). The bacterial amplicon sequence was then sequenced by the Illumina MiSeq platform (Illumina, San Diego, CA, USA). The sequenced data processing was operated using the Divisive Amplicon Denoising Algorithm 2 (DADA2) R package (Callahan et al., 2016). The representative amplicon sequence variant (ASV) sequences were identified by the SILVA (v183) and classified into specific bacterial taxonomy. Single sample diversity analysis was accomplished by Alpha diversity index using QIIME: ACE, Chao1, Shannon, and Simpson.

### Functional gene prediction

Phylogenetic investigation of communities by reconstruction of unobserved states (PICRUSt), a bioinformatics tool to predict the metagenome gene’s functional content using 16S rRNA genes, was applied to obtain an overview of the metagenomic contribution of the colonic content and mucosal bacterial community. Predictions were made for genes in databases, including the metagenome inference of Kyoto Encyclopedia of Genes and Genomes (KEGG).

### Statistical analyses

The independent-sample *t*-test was employed to calculate the differences in fermentation parameters between groups. The two-way repeated analysis of variance (ANOVA) evaluation was used to calculate the difference in diversity index between groups. Analysis of similarities (ANOSIM) of bacterial community based in unweighted unifrac distance among the three groups was performed by a principal coordinate analysis (PCoA) using the SIMCA-P (version 14.0) software package (Umetrics, Umea, Sweden). The taxonomy data from the experiment were initially organized in Excel and then statistically calculated by the SPSS software package v.25 (SPSS Inc., Chicago, IL, USA). The normality of the distribution of variables was tested using the Shapiro Wilk. When the variable distributions were assumed normal, the independent samples *t*-test procedure was used; otherwise, the Kruskal-Wallis procedure was used. *P* < 0.05 indicates a significant difference, *P* < 0.01 indicates a significantly difference.

## Results

### Colonic pH, concentrations of volatile fatty acids, and epithelium morphological structure in weaned lambs

As shown in Table 1, compared with CON, colonic pH in CM showed a decreasing trend (*P* = 0.069), and a significant increase in the concentration of total VFA (*P* = 0.035). Between the CM and the CG groups, there was no significant difference in colonic pH and the proportion of VFAs.

**TABLE 1:** The effect of CON, CM, and CG on colonic fermentation parameters of weaned lambs^1^.

Item	CON^3^	CM^3^	CG^3^	*P*-value	
				
				CON vs. CM	CM vs. CG
Colonic pH	6.80 ± 0.05	6.45 ± 0.17	6.59 ± 0.07	0.069	0.418
Total VFA^2^, mM	73.56 ± 6.74	102.69 ± 9.88	89.97 ± 3.80	0.035	0.228
Acetate, mol/100 mol of VFA	74.11 ± 1.10	69.25 ± 2.96	71.13 ± 1.49	0.172	0.588
Propionate, mol/100 mol of VFA	16.07 ± 0.99	16.89 ± 0.80	15.99 ± 0.86	0.535	0.460
Isobutyrate, mol/100 mol of VFA	1.11 ± 1.52	0.81 ± 0.17	0.81 ± 0.13	0.224	0.980
Butyrate, mol/100 mol of VFA	6.48 ± 0.62	11.26 ± 2.92	10.00 ± 1.03	0.165	0.698
Isovalerate, mol/100 mol of VFA	1.00 ± 1.74	0.75 ± 0.18	0.78 ± 0.14	0.331	0.881
Valerate, mol/100 mol of VFA	1.23 ± 0.13	1.03 ± 0.12	1.29 ± 0.18	0.285	0.258

^1^The values shown are means ± SEM (standard error of the mean); *P* < 0.05 indicated that mean values were significantly difference.

^2^VFA, volatile fatty acids.

^3^CON = maize meal low-grain diet, CM = maize meal high-grain diet, CG = whole maize high-grain diet.

**FIGURE 1:**
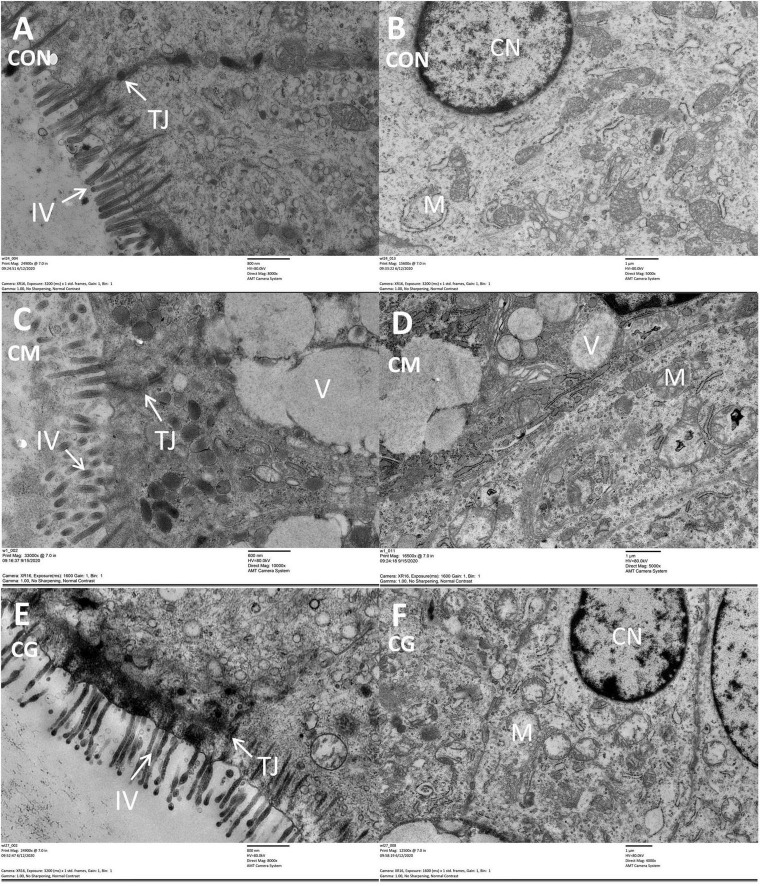
The histology of colon tissue among low-grain fed (CON), maize meal high-grain fed (CM), and whole maize high-grain fed (CG) lambs. Comparison of colonic epithelial ultrastructure of junctional complexes among the CON [**(A)** scale bar = 800 nm], CM [**(C)** scale bar = 600 nm], and CG [**(E)** scale bar = 800 nm]. The colonic epithelial ultrastructure in representative CON [**(B)** scale bar = 1 μm], CM [**(D)** scale bar = 1 μm], and CG [**(F)** scale bar = 1 μm]. TJ, tight junction; IV, intestinal villus; CN, cell nucleus; M, mitochondria; V, vacuole.

Compared with CON group, the intestinal villus and colonic epithelial mitochondria were damaged and vacuoles appeared in the colonic epithelial cell in CM group (Figure 1). Although the same high-gain level diet was used, the morphology of the colonic epithelium was significantly improved in CG compared to CM, suggesting that the whole maize form could protect the colonic environment in a high-gain diet feeding.

### Colonic content microbiota in weaned lambs

#### Diversity of the colonic content microbiota

**FIGURE 2:**
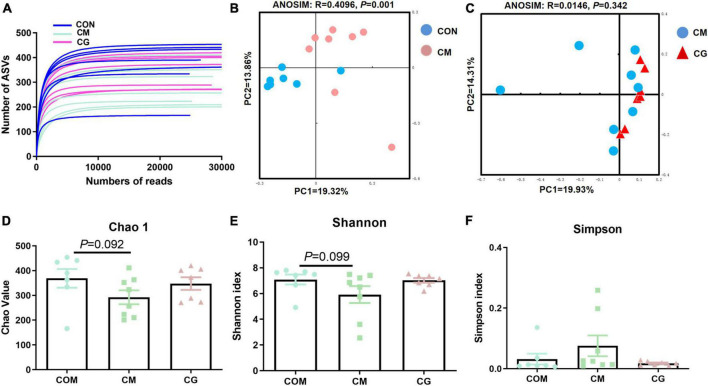
Effects of low-grain fed (CON), maize meal high-grain fed (CM), and whole maize high-grain fed (CG) on the diversity of colonic content bacterial structures. **(A)** Bacterial dilution curve of colonic content bacterial community. Horizontal coordinate: the amount of randomly selected sequencing data; vertical coordinate: the number of observed amplicon sequence variants (ASVs). **(B)** Unweighted UniFrac principal coordinate analysis (PCoA) of bacterial communities in the colonic content between the CON and CM. **(C)** Unweighted UniFrac principal coordinate analysis (PCoA) of bacterial communities between the CM and CG. Effects of the CON, CM, and CG on the diversity of colonic content bacterial community: Chao1 **(D)**, Shannon **(E)**, Simpson **(F)**.

The bacterial rarefaction profile of colon content flattened, indicating that the sequencing depth and data volume were sufficient to cover most microbes (Figure 2A). The microbial composition was significantly distinct in CON and CM (ANOSIM, *R* = 0.4096, *P* = 0.001) but not in CM and CG (ANOSIM, *R* = 0.0146, *P* = 0.342) (Figures 2B,C).

As shown in Figures 2D,E, there was a decreasing trend of Chao 1 (*P* = 0.092) and Shannon’s estimate (*P* = 0.099) for colonic content in CM compared to CON. The diversity index of the content microbiota did not significantly differ between the CM and the CG (*P* > 0.05), despite the CG having a higher number of Chao 1, Shannon indexes, and lower Simpson index (Figures 2D–F). These findings indicated that the colonic content microbial diversity within a high-gain diet could not be considerably improved by whole maize form.

#### Composition of the colonic content microbiota

**FIGURE 3:**
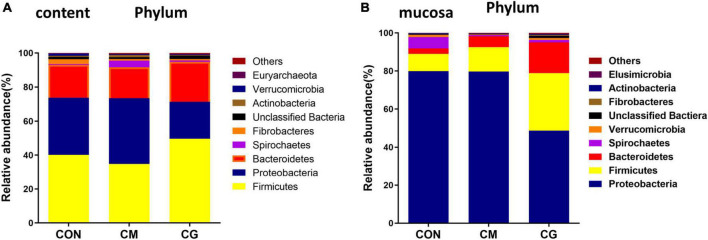
Effects of low-grain fed (CON), maize meal high-grain fed (CM), and whole maize high-grain fed (CG) on the relative abundance of phylum level in colonic content and mucosa. **(A)** The distribution of phylum for each sample in colonic content. **(B)** Distribution of the phylum in colonic mucosa among the CON, CM, and CG groups.

There were nine common phyla detected in the colonic content samples among the three groups: Firmicutes, Proteobacteria, Bacteroidetes, Spirochaetae, Fibrobacteres, Unclassified Bacteria, Actinobacteria, Verrucomicrobia, and Euryarchaeota (Figure 3A). The relative abundance of Candidatus, Saccharibacteria, and Unclassified Bacteria in the CM were substantially lower than those in the CON (*P* < 0.05).

**FIGURE 4:**
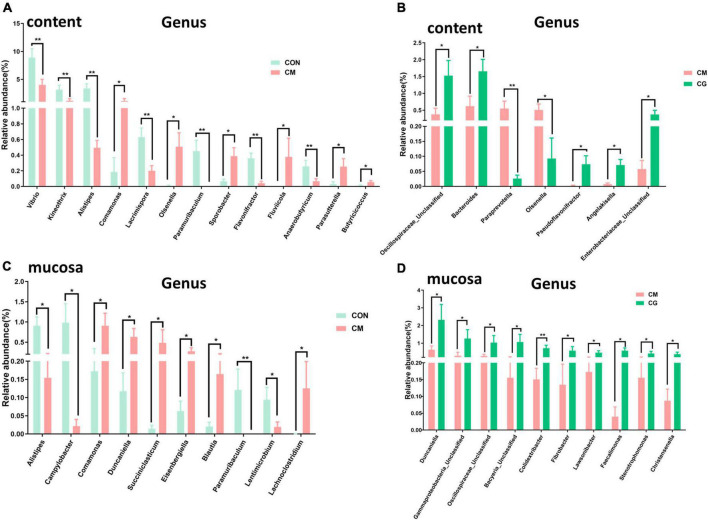
Effects of low-grain fed (CON), maize meal high-grain fed (CM), and whole maize high-grain fed (CG) on the genus level bacteria in colonic content and mucosa. **(A)** The comparison of genus level bacterial relative abundance in colonic content between the CON and CM group. **(B)** The differences of bacterial relative abundance in colonic content between the CM and CG group. **(C)** Effects of low-grain fed (CON), and maize meal high-grain fed (CM) on colonic mucosal bacterial relative abundance. **(D)** The colonic mucosal bacteria respond to the maize meal high-grain fed (CM) and whole maize high-grain fed (CG). *P < 0.05 indicated that mean values were significantly difference. **P < 0.01 indicated that mean values were greatly significantly difference.

The relative abundance of 183 genera was more than 0.1 %. In Figures 4A,B, some of the genera with notable differences are depicted. Compared with the CON, the relative abundance of *Vibrio*, *Kineothrix*, *Alistipes*, *Lacrimispora*, *Paramuribaculum*, *Flavonifractor*, and *Anaerobutyricum* in colonic content of the CM were highly significantly decreased (*P* < 0.01); the relative abundance of *Comamonas*, *Olsenella*, *Sporobacter*, *Fluviicola*, *Parasutterella*, and *Butyricicoccus* were significantly increased (*P* < 0.05). Compared with the CM, the CG decreased the relative abundance of *Paraprevotella* (*P* < 0.01) and *Olsenella* (*P* < 0.05) while increased the relative abundance of Unclassified Oscillospiraceae, *Bacteroides*, *Pseudoflavonifractor*, *Angelakisella*, and Unclassified Enterobacteriaceae significantly (*P* < 0.05). These findings suggested that the whole maize form throughout a long-term high-gain diet restored the alterations in the content microbial community.

#### Functional prediction of colonic content microbiota

**FIGURE 5:**
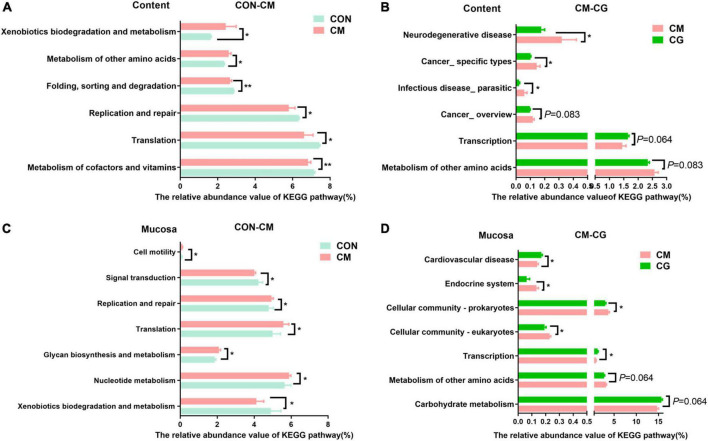
Effects of low-grain fed (CON), maize meal high-grain fed (CM) and whole maize high-grain fed (CG) on the function of colonic content and mucosal bacteria. **(A)** Kyoto Encyclopedia of Genes and Genomes (KEGG) pathways of colonic content bacteria in the CON and CM group. **(B)** KECG pathways of colonic content bacteria in the CM and CG group. **(C)** Microbial KEGG pathway of colonic mucosal bacteria in the CON and CM group. **(D)** Microbial KEGG pathway of colonic mucosal bacteria in the CM and CG group. *P < 0.05 indicated that mean values were significantly difference. **P < 0.01 indicated that mean values were greatly significantly difference.

The functional prediction of colonic content microbiota was shown in Figures 5A,B. Compared with the CON, the relative abundance of genes encoding metabolism of cofactors and vitamins (*P* = 0.004), folding, sorting and degradation (*P* = 0.001), translation, replication, and repair (*P* = 0.037) in colonic microbiota of the CM were significantly decreased. In contrast, the relative abundance of genes encoding metabolism of other amino acids (*P* = 0.037), xenobiotics biodegradation and metabolism (*P* = 0.015) were significantly increased. Additionally, the colonic microbiota of the CG showed substantially lower relative abundance of genes encoding infectious disease_ parasitic (*P* = 0.049), cancer_ specific kinds (*P* = 0.049), and neurodegenerative disease (*P* = 0.037) compared to the CM, demonstrating how the whole maize form improved colonic microbiota’s capability to combat infection under high-gain diet feeding.

### Colonic mucosal microbiome of weaned lambs

#### Diversity of colonic mucosal microbiota

**FIGURE 6:**
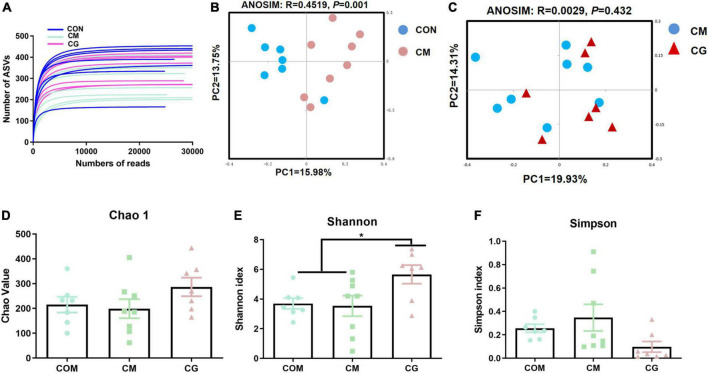
Effects of low-grain fed (CON), maize meal high-grain fed (CM), and whole maize high-grain fed (CG) on the diversity of colonic mucosal bacterial structures. **(A)** Bacterial dilution curve of colonic mucosal bacterial community. Horizontal coordinate: the amount of randomly selected sequencing data; vertical coordinate: the number of observed amplicon sequence variants (ASVs). **(B)** Unweighted UniFrac principal coordinate analysis (PCoA) of bacterial communities in the colonic mucosa between the CON and CM. **(C)** Unweighted UniFrac principal coordinate analysis (PCoA) of bacterial communities between the CM and CG. Effects of the CON, CM, and CG on the diversity of colonic mucosal bacterial community: Chao1 **(D)**, Shannon **(E)**, Simpson **(F)**. *P < 0.05 indicated that mean values were significantly difference.

The rarefaction curves of colonic mucosal microbiota tended to be flat, indicating reasonable sampling and sufficient sequencing depth to reflect the bacterial community in the samples (Figure 6A). Figures 6B,C show the bacterial community profiles in the colonic mucosa of the CON, the CM, and the CM and the CG. The results of the Bray-Curtis metric revealed clear dissimilarities between CON and CM (ANOSIM, *R* = 0.4519, *P* = 0.001), while there was no significant segregation between CM and CG (ANOSIM, *R* = 0.0029, *P* = 0.432).

As shown in Figures 6D–F, no significant difference was observed in the colonic mucosal bacterial community diversity index between CON and CM (*P* > 0.05). And compared with the CON and the CM, the Shannon index of colonic mucosa in the CG was significantly increased (*P* < 0.05), indicating a higher microbial diversity in the whole maize from diet feeding.

#### Composition of the colonic mucosal microbiota

The common phyla detected in the colonic mucosa samples of the three groups were: Proteobacteria, Firmicutes, Bacteroidetes, Spirochaetes, Verrucomicrobia, Unclassified Bactiera, Fibrobacteres, Actinobacteria, and Elusimicrobia (Figure 3B). Compared with the CM, the relative abundance of Proteobacteria in the CG was significantly decreased (*P* < 0.05), and the relative abundance of Firmicutes, Fibrobacteres, and Unclassified Bactiera were significantly increased (*P* < 0.05).

At least one set of relative abundance greater than 0.1 % was found in 159 genera. It can be seen from Figure 4C that compared with the CON, the relative abundance of *Paramuribaculum* in the colonic mucosa of the CM was highly significantly decreased (*P* < 0.01); the relative abundance of *Alistipes* and *Campylobacter* decreased significantly (*P* < 0.05); and the relative abundance of *Comamonas*, *Duncaniella*, *Succiniclasticum*, *Eisenbergiella*, *Blautia*, and *Lachnoclostridium* were dramatically increased (*P* < 0.05). And as shown in Figure 4D, compared with the CM, the relative abundance of *Colidextribacter* in the colonic mucosa of the CG was highly considerably increased (*P* < 0.01); the relative abundance of *Duncaniella*, Unclassified Gammaproteobacteria, Unclassified Oscillospiraceae, Unclassified Bacyeria, *Fibrobacter*, *Lawsonibacter*, *Faecalimonas*, *Stenotrophomonas*, and *Christensenella* were pronouncedly increased (*P* < 0.05). These findings demonstrated that the maize particle size significantly influenced the mucosal microbial population under a prolonged high-gain diet.

#### Functional prediction of colonic mucosal microbiota

Figures 5C,D show the functional prediction of colonic mucosal microbiota. Compared with the CON, the relative abundance of genes encoding nucleotide metabolism (*P* = 0.021), glycan biosynthesis and metabolism (*P* = 0.015), translation (*P* = 0.028), replication and repair (*P* = 0.021), cell motility (*P* = 0.049) in the CM were significantly increased, while the relative abundance of genes encoding xenobiotics biodegradation and metabolism (*P* = 0.049), signal transduction (*P* = 0.021) were significantly decreased. And compared with the CM, the CG significantly increased the relative abundance of genes encoding transcription (*P* = 0.028) and cardiovascular disease (*P* = 0.037). In contrast, the relative abundance of genes encoding cellular community_eukaryotes (*P* = 0.021), cellular community_ prokaryotes (*P* = 0.049), and endocrine system (*P* = 0.037) were significantly decreased.

## Discussion

### Colonic pH, concentrations of volatile fatty acids, and epithelium morphological structure

In this experiment, the CM tended to have lower colonic pH and greater concentrations of VFAs than the CON, which is similar to the findings of earlier studies on the goat colon (Ye et al., 2016). When a high-grain diet was fed for a long time, excessive carbohydrates could escape from the rumen fermentation and enter the ruminant’s intestine (Metzler-Zebeli et al., 2013). Microbes could largely use these fermented carbohydrates in the colon to produce short-chain fatty acids that will likely lower the colonic pH and cause hindgut acidosis as well as inflammation (Tao et al., 2015). The CM and CG did not differ substantially in terms of colonic pH, total VFAs, or any specific short-chain fatty acids.

The surface layer epithelium, intercellular tight junction, and cell mitochondria of the CM, however, were significantly damaged compared to the CON during microscopic observation of the lamb colonic epithelium, which is in line with the previous investigation in the goat colon (Ye et al., 2016). And the colonic epithelium of the CG was relatively intact. It was speculated that the feeding form of whole maize mitigated the extent of colonic epithelial damage from a high-grain diet and was beneficial for further nutrient absorption and immune function for the lambs.

### Adaptability of the colonic content microbiota in weaned lambs

The gastrointestinal microbiota is vital for ruminants and plays an indispensable role in the digestion and absorption of feed and gastrointestinal development (Yanez-Ruiz et al., 2015). In the present study, we investigated the adaptive response of colonic content microbiota with different particle sizes maize feeding through high-throughput sequencing. The dominant phyla of the three groups were Firmicutes, Bacteroidetes, and Proteobacteria, consistent with the bacterial community previously found in the colonic tissue of male goats (Ye et al., 2016). Several previous studies have shown that phyla Firmicutes and Bacteroidetes function in fermentation, metabolism, and degradation of carbon sources, oligosaccharides (Wetzels et al., 2015), protein and amino acids (Tang et al., 2005).

In comparison to CON, *Parasutterella*, *Comamonas*, and *Butyricicoccus* had considerably greater relative abundance in CM. Although genera *Parasutterella* has been reported in the gastrointestinal tract of cattle, its precise function is yet unclear (Kim and Wells, 2016; Zhang et al., 2017). Previous studies have shown that *Comamonas* and *Butyricicoccus* are important butyrates and hydroxybutyric acid producers (Ys and Yd, 1994; Pryde et al., 2002). And genera *Comamonas* has also been found in the rumen of severe feed restriction and tissue-associated community of heifers (Petri et al., 2013; Fan et al., 2018). High-grain diets allow more soluble carbohydrates to enter the colon as fermentation substrates; therefore, the relative abundance of these butyrate-producing microbiota is elevated.

The relative abundance of the genera *Bacteroides* and *Angelakisella* were substantially greater in the CG compared to the CM. It has been shown that genera *Bacteroides* involve the degradation of starch and fiber (Dias et al., 2018) and can ferment various monosaccharides (Holdeman et al., 1976). Genera *Angelakisella* has been isolated from the human ileum (Mailhe et al., 2017) and is thought to be able to regulate the short-chain fatty acids production (Qiu et al., 2020). The enhancement of these fermentation-associated microbiotas has the opportunity to facilitate energy production and nutrient utilization in the colon.

It was noteworthy that the relative abundance of *Olsenella* was significantly higher in CM than in the CON and CG groups. Petri et al. (2013) reported that *Olsenella* dominates in beef cattle during subacute ruminal acidosis by pyrosequencing techniques. We hypothesize that the feeding strategy of whole maize might lessen the likelihood of acidosis brought on by high-grain diets since the relative quantity of *Olsenella* is lower in CG. However, our measured colonic fermentation profiles did not reveal any discernible differences in VFAs, pH, etc., between the CM and the CG, so follow-up studies are needed to explore the effect of maize particle size on colonic fermentation.

### Adaptability of the colonic mucosal microbiota in weaned lambs

The colonic epithelium, covered with mucosa-associated bacteria, serves as the host’s physical and immunological barrier (Kostic et al., 2013). Changes in the relative abundance of these microbiotas may affect the colonic immune functions and even the host’s health status (Donaldson et al., 2016). In this experiment, we explored the adaptive response of lamb colonic mucosal microbiota to various feeding regimens. Proteobacteria, Firmicutes, and Bacteroidetes were the most prevalent phyla in all three groups. Phyla Firmicutes and Bacteroidetes were also previously shown to be the dominant phyla in the colonic mucosal flora of goats (Ye et al., 2016) and calves (Malmuthuge et al., 2014). Bacteria belonging to phyla Bacteroidetes and Firmicutes can promote the fermentation of plant-based fibrous polysaccharides and regulate the absorption of proteins and other nutrients, which is vital for the host health (Liu et al., 2017).

In line with variations in the colonic content, the relative abundance of the acetate producer *Alistipes* at the genus level was much lower in the CM than it was in the CON (Oliphant and Allen-Vercoe, 2019). As an anaerobic bacterium, *Alistipes* was found mainly in the intestines of healthy animals (Shkoporov et al., 2015). It can regulate intestinal inflammation and relieve disease through short-chain fatty acids (Harrisham et al., 2017). However, some studies showed that *Alistipes* might be associated with colorectal cancer (Moschen et al., 2016), cardiovascular disease (Zuo et al., 2019) and hepatic encephalopathy (Chang et al., 2019). So different species of *Alistipes* may play various roles in host’s health (Parker et al., 2020). Thus, further studies are needed to clarify the mechanism. Meanwhile, the relative abundance of genera *Campylobacter* was significantly reduced in the CM than that in the CON. As a common gastrointestinal pathogen, microbes belonging to the genera *Campylobacter* are frequently found in the gastrointestinal tract of ruminants, including cattle and sheep, the relative abundance of which can be as high as 20% in lamb rumen (Diker et al., 1990; Guyard-Nicodeme et al., 2016). Numerous studies have revealed that certain bacteria in this genus are susceptible to low pH levels and that some members of this genus have been linked to localized colonic inflammation in people or animals (Blaser et al., 1980; Grayson et al., 1989; Russell et al., 1993; Chen et al., 2007). In this experiment, there was a decreased trend of pH in the colon of the CM compared to the CON, consistent with the conclusion mentioned above. We also found that the relative abundance of butyrate-producing genera *Succiniclasticum* and *Comamonas* were significantly elevated in the CM compared to the CON (Ys and Yd, 1994; Koike and Kobayashi, 2009). Butyrate has been shown in numerous studies to demonstrate beneficial effects on the repair of the rumen epithelium (Poller et al., 2004; Zhang et al., 2018), the development of the cecum epithelium (Kien et al., 2007; Malhi et al., 2013), and the prevention of the occurrence of colonic inflammation (Inan et al., 2000). The gut mucosa-associated microbiota can change their densities to adapt to the altered host’s internal environment (Blaser and Kirschner, 2007). Combined with the results of colon damage observed under transmission electron microscopy, we speculate that the increased relative abundance of genera *Succiniclasticum* and *Comamonas* in the CM may be reactive response of lambs to avoid more severe damage to the colonic epithelium.

Furthermore, the relative abundance of genera *Duncaniella* was significantly increased in the CM relative to the CON, while it further rose considerably in the CG. Although research has shown that the *Duncaniella* genus predominates in the mouse gut, its precise role is yet unknown (Miyake et al., 2020). The relative abundance of genera *Christensenella*, *Lawsonibacter*, and *Coldextribacter* in the CG were significantly higher than that in the CM. Genera *Christensenella* is a common resident in the rumen and plays an essential role in maintaining the structure and function of the gastrointestinal tract (Jenkins et al., 2015). Genera *Lawsonibacter* can produce butyrate (Sakamoto et al., 2018), which is linked to the prevention of colitis and colorectal cancer (Mcintyre et al., 1993; Archer et al., 1998). Genera *Colidextribacter* has been reported to reduce tissue damage and inflammation through modulating signaling pathways (Mager et al., 2020; Guo et al., 2021). All three genera are associated with body health, and the higher relative abundance shows that the whole maize feeding pattern may have favorable effects on the development and function of the intestinal mucosa. Our microscopic observations of the colonic epithelium also indicated that the colonic epithelium in the CM was severely damaged, whereas the CG was relatively intact and similar to the CON. It was proved again that whole maize could alleviate the damage of colon epithelium caused by high-grain diets and promote the development of lamb colon.

### Functional prediction of colonic content and mucosal microbiome

Intestinal bacterial community plays an important role in the immune system and maintaining the body’s health (Hooper et al., 2012). According to earlier research, diet is a key factor in the interaction between the host genome and the microbiota (Holmes et al., 2012). PICRUSt was also used to investigate the metagenomic and potential functions of the colonic content and mucosal microbiota in lambs in this experiment. We found considerably higher relative abundance of genes encoding degradation and metabolism in the colonic content microbiota of the CM compared to the CON, which is likely a result of larger quantity of degradable carbohydrates entering the colon from the high-grain diet. Meanwhile, we discovered a significant decrease in genes encoding various diseases in the colonic microbiota of the CG than that in CM. This alteration in the relative abundance of digestion-related genes hypothesized that feeding the whole maize high-grain diet would reduce the incidence of intracolonic diseases resulting from the high-grain diet.

### Adaptation of differences between colonic content and mucosa microbiome to different grain sizes

In this experiment, 183 genera of content microbiota were detected, including 7 genera with significant difference in the CM and the CG; and 159 genera of mucosal microbiota were detected, including 18 genera with significant difference between the CM and the CG. The results revealed that the impact of various maize forms on microbiota in lamb colonic mucosa was more substantial than that in colonic content, which may be related to the two colonic areas’ various activities. The microbiota in the colonic content plays a vital role in the digestion and metabolism of nutrients. In contrast, the colonic mucosal microbiota, as an important component of the colonic epithelial barrier, is mainly related to the maintenance of the colonic epithelial barrier (Nagy-Szakal et al., 2012; Kostic et al., 2013). Pryde et al. (1999) found that microbiota colonizing in the pig’s colonic wall differed strongly from that in the colonic and cecal lumen. Microbiota in the intestinal wall of animals plays a potential function in the interaction with the host (Bengmark, 1996); hence it is hypothesized that the changes made by microbiota in the intestinal wall in response to external stimuli are more pronounced than in the intestinal lumen. Second, the more sensitive response of colonic mucosal microbiota to the maize form may be related to the colonic structure. The surface of colonic mucosa is smooth and is dwelled by numerous microbiota. The microbiota in the goats’ hind gut mucosa has previously been demonstrated to be extremely responsive to diet (Liu et al., 2014; Ye et al., 2016). Therefore, the fermentation environment in the colon follows when the maize form changes, which will further lead to the relative abundance changes of the bacterial community in the colonic mucosa to achieve homeostatic reconstruction. Third, having more carbohydrates in the colon leads to an increase in the osmotic pressure of the colon wall, which might impair the structure of the colonic mucosa by increasing the permeability of the colonic epithelium (Gressley et al., 2011), and further leads to the altered mucosal microbial community to a deeper extent than the content microbiota. The increased permeability of the intestinal tract was speculated to cause the absorption of toxins such as histamine in the content (Gressley et al., 2011). We experimentally observed that a prolonged high-grain diet significantly led to damage to the colonic epithelium, and the damage was mitigated in the colonic mucosa of lambs fed a whole-grain diet.

## Conclusion

**FIGURE 7:**
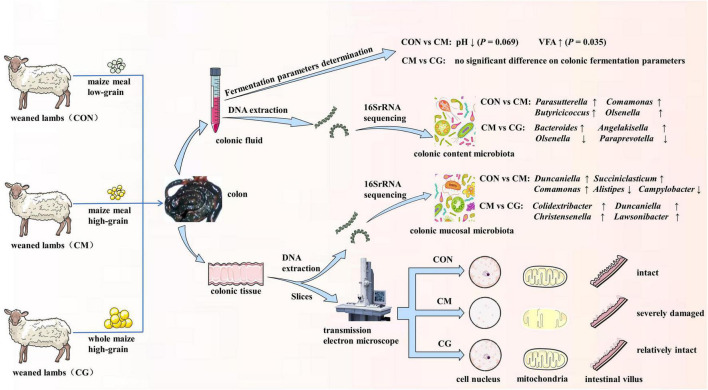
The schematic diagram of experimental design and main results in this study.

We found that feeding weaned lambs a whole-maize, high-grain diet may reorganize the colon’s bacterial population and restore some of harm brought on by a high-grain diet (Figure 7). Furthermore, microbiota associated with the colonic mucosal region responded greater than those in the colonic content to different maize forms. This study provides theoretical guidance for the application of whole maize high-grain diet in practical production.

## Data availability statement

The sequencing data have been deposited in the NCBI Sequence Read Archive (SRA) database under the accession code: SRP387601.

## Ethics statement

The animal experiment was approved by the Animal Protection and Use Committee of Nanjing Agricultural University (Authorisation SYXK(Su)2019-0074) and was performed following the Regulations for the Administration of Affairs Concerning Experimental Animals (The State Science and Technology Commission of P. R. China, 1988).

## Author contributions

CC and YY carried out most of the experiment including animal care, VFA analyses, DNA isolation, and histological measurements. CC and GB were responsible for 16S rRNA high throughput data processing, analyses, interpretation, and manuscript preparation. GB contributed to the conception of the project. All authors contributed to the article and approved the submitted version.
