# Supplementary material for: Effects of whole maize high-grain diet feeding on colonic fermentation and bacterial community in weaned lambs
Source: Front Microbiol. 2022 Dec 8;13:1018284. doi: 10.3389/fmicb.2022.1018284 (PMC9772272; doi:10.3389/fmicb.2022.1018284)
Supplement: Supplementary file 1 [file Table_1.DOCX]

**Supplementary Table 1.** *Ingredient and chemical composition of the diet*

| **Item** | **CON** | **CM** | **CG** |
| --- | --- | --- | --- |
| **Ingredient (% DM)** |  |  |  |
| Oat hay | 52.00 | 20.00 | 20.00 |
| Alfalfa hay | 18.00 | 10.00 | 10.00 |
| Maize | 19.20 | 50.40 | 50.40 |
| Soybean meal | 6.70 | 15.00 | 15.00 |
| Limestone meal | 0.50 | 1.30 | 1.30 |
| Salt | 0.70 | 0.50 | 0.50 |
| Dicalcium phosphate | 0.90 | 0.80 | 0.80 |
| Mineral and vitamin mixture^1^ | 2.00 | 2.00 | 2.00 |
| **Component (% DM)** |  |  |  |
| Metabolic energy (MJ/kg DM) | 8.92 | 10.16 | 10.16 |
| Crude protein | 13.80 | 16.17 | 17.77 |
| Crude fat | 3.01 | 3.37 | 3.62 |
| Crude fiber | 20.89 | 8.62 | 8.62 |
| Starch | 13.61 | 35.71 | 33.83 |
| Neutral detergent fiber | 40.55 | 22.52 | 23.35 |
| Crude ash | 6.89 | 7.71 | 7.08 |
| Calcium | 0.95 | 0.95 | 0.95 |
| Phosphorus | 0.44 | 0.44 | 0.44 |

^1^ Per kg premix contains: Fe 3.0 g, Zn 5.0 g, Cu 0.5 g, Mn 3.0 g, Co 0.1 g, I 50 mg, Se 40 mg, vitamin A 500,000 IU, vitamin D 50,000 IU, and vitamin E 2,000 IU.

^2^ CON = maize meal low-grain diet, CM = maize meal high-grain diet, CG = whole maize high-grain diet

^3^Metabolic energy, crude fat, calcium, and phosphorus were calculated value (MOA, 2004). Crude protein, starch, neutral detergent fiber, acid detergent fiber, and crude ash were measured using standard methods of AOAC (2007).
